# Tracking pleural sliding motion to assess lung overdistention using an open source algorithm: a proof-of-concept study on lung ultrasound scans

**DOI:** 10.1186/s13054-025-05742-8

**Published:** 2026-01-22

**Authors:** Andrea Costamagna, Marry R. Smit, Emanuele Pivetta, Paolo Persona, Paolo Navalesi, Luigi Pisani, Marcus J. Schultz, Luca Brazzi, Vito Fanelli, Pieter R. Tuinman, Lieuwe D.J. Bos

**Affiliations:** 1 Department of Surgical Sciences, University of Turin, C.so Dogliotti 14, Turin, 10126 Italy; 2 Department of Intensive Care, Amsterdam University Medical Centers, Amsterdam, the Netherlands; 3 Department of Medical Sciences, University of Turin, Turin, Italy; 4 Neuro and Trauma Critical Care Unit, Verona University Hospital, Verona, Italy; 5 Medicine Department (DIMED), University of Padua, Padua, Italy; 6 University of Bari ’Aldo Moro’, Bari, Italy; 7 University of Amsterdam, Amsterdam, the Netherlands

**Keywords:** Lung ultrasound, Motion tracking, Detection algorithms, Pleural line, Lung sliding, Quantitative lung ultrasound, Overdistention, Acute respiratory distress syndrome

## Abstract

**Background:**

Pleural line (PL) movement, assessed by lung ultrasound, is crucial for the detection of pneumothorax and might also indicate overdistention, but research is limited by the lack of a quantitative tool. We set out to answer two research questions: can PL movement be quantified using open-source motion tracking software, and can PL movement be used to identify overdistention? We hypothesize that motion tracking of the PL is feasible and represents an accurate estimation of lung sliding.

**Methods:**

Lung ultrasound video clips from three patient groups were used: (1) healthy volunteers during expiratory hold maneuvers (functional residual capacity) and quiet breathing, (2) ICU patients, blindly assessed for lung sliding (absent, doubtful, evident but limited or evident and extensive) and (3) Severe COVID-19 viral pneumonia patients undergoing PEEP titration and electrical-impedance tomography. Open-source software that implements the “Channel and Spatial Reliability Tracking” tracker algorithm was used for motion tracking, identifying the PL at three points and a soft tissue reference. Each motion-time curve was subsequently smoothed and normalized to account for soft tissue displacement. The maximum lateral movement on the transversal plane among the three normalized PL landmarks defined PL movement.

**Results:**

In 143 video clips from 7 healthy individuals, PL movement increased from functional residual capacity (1.2 ± 0.6 mm) to quiet breathing (5.4 ± 2.5 mm; *p* < 0.01). In 336 video clips from 40 ICU patients, PL movement increased from absent (2.7 ± 1.2 mm) to extensive lung sliding (14.7 ± 5.8 mm; *p* < 0.01). Ordered logistic regression predicted Absent sliding with 71% balanced accuracy, with motion tracking correctly identifying all cases and no patients without lung sliding misclassified as extensive, based on visual inspection of the pleural line. In 358 video clips from 30 patients undergoing PEEP titration, there was an association between overdistention quantified by electrical-impedance tomography and PL movement (Spearman-rho=−0.6). PL movement decreased from low to high PEEP levels (*p* < 0.01).

**Conclusions:**

Pleural line motion tracking is feasible and provides quantitative insight into pleural movement based on data from healthy volunteers and visual inspection of images from ICU patients. Moreover, pleural line movement allows accurate assessment of overdistention during mechanical ventilation when compared with electrical-impedance tomography.

**Graphical abstract:**

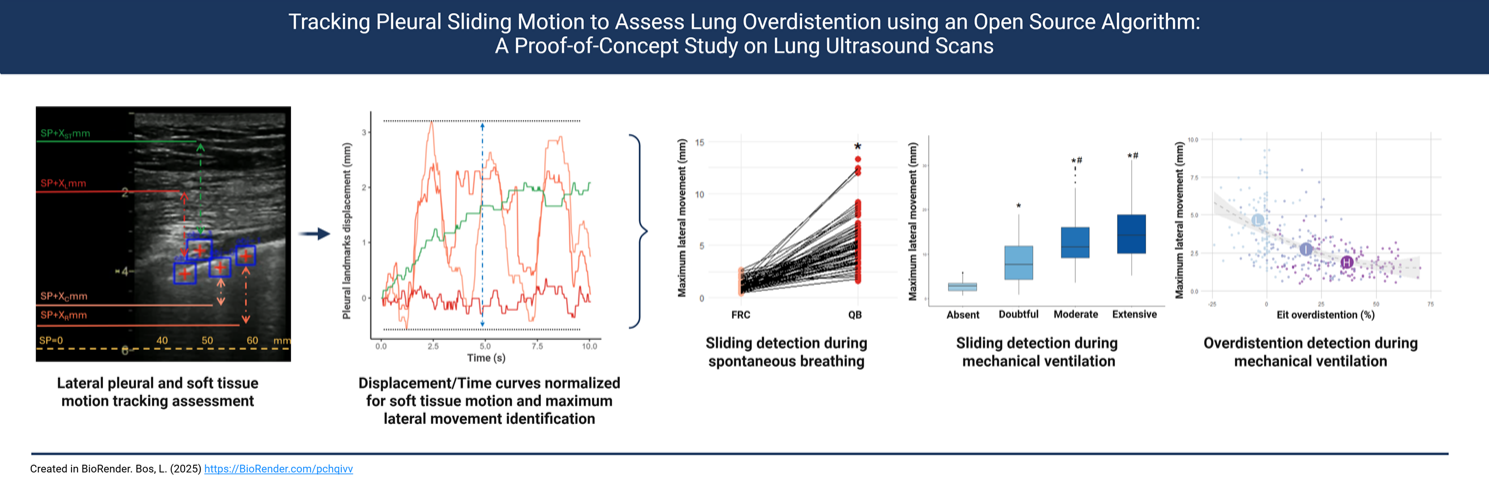

**Supplementary Information:**

The online version contains supplementary material available at 10.1186/s13054-025-05742-8.

## Background

Acute respiratory distress syndrome (ARDS) is common in intensive care units (ICU) and is associated with a mortality around 40%. Mechanical ventilation is a life-saving intervention but poses the risk of ventilator induced lung injury (VILI). This is particularly important in patients with ARDS, as alveolar filling and atelectasis redirect the mechanical power of mechanical ventilation to a smaller sized lung. Overdistention is crucial to monitor in this so-called baby-lung to prevent and limit the extent of VILI [1].

Chest computed tomography is the gold standard for assessing recruitment and overdistention but is impractical for routine use due to transport risks and radiation exposure [2]. Electrical impedance tomography (EIT) offers an alternative but requires ventilator titration and remains a research tool [3]. Lung Ultrasound (LUS) has emerged as a widely available, reliable bedside tool for diagnosis and monitoring in critical care settings [4–9]. LUS relies on artifacts to assess lung recruitment, but cannot detect lung overdistention since any gas-filled cavity generates an A-line artifact [10–12].

The quantification of pleural line (PL) movement, or ‘lung sliding’, has been suggested as a marker of overdistention [13, 14]. But while qualitative PL evaluation is used for specific conditions such as pneumothorax and pleural effusion, the ultrasonographic quantification of PL movement is still limited [7]. Strain, a speckle-tracking based measurement originated from echocardiography, has recently been proposed to quantify PL movement [15, 16]. Pleural strain measurement based on ultrasound elastography and speckle-tracking is generally feasible [16–18], and is clinically correlated to pneumothorax [19–21]. Recent findings suggest that overdistention measured by EIT also results in decreased lung sliding [22].

Various speckle-tracking algorithms are available, and each has limitations for widespread application in LUS. Most commercial versions are patented for specific machines, tailored to cardiac tissue, and depend on high-quality native formats, whereas LUS is often performed with basic equipment. In addition, the lung is not a solid structure; consequently, ultrasound speckle patterns, although present, are unreliable, unpredictable, and unsuitable for speckle-tracking based strain analysis as it is currently understood. Motion tracking (MT) techniques, although sharing some similarities with the foundational aspects of speckle tracking, allow a simple and direct evaluation of the movement of a given point of a structure over time, making MT suitable for studying the kinetics of the two pleural layers during the respiratory cycle, independently of the characteristics of the underlying parenchyma. Open-source MT algorithms that can operate on any type of video format are promising and could facilitate the implementation, training, and development of artificial intelligence models for prospective testing [23–25]. Open Source Computer Vision Library (OpenCV) is an open-source library which provides a robust framework for implementing real-time image processing techniques, making it well-suited even for medical imaging applications [24, 26].

Using data from healthy volunteers and ICU patients, we set out to answer two research questions: can PL movement be quantified using open-source MT software, and can PL movement be used to identify overdistention? We hypothesize that motion tracking of the PL is feasible and represents an accurate estimation of lung sliding. More specifically, the tracked motion of the pleural line is consistent with the breathing pattern in healthy volunteers, correlates with the magnitude of pleural line movement assessed by visual inspection in ICU patients, and shows an inverse correlation with the occurrence of overdistention in patients with Severe COVID-19 viral pneumonia during mechanical ventilation.

## Methods

### Study population

The study was based on the secondary analysis of LUS videos obtained in previous studies:

- Unito cohort: Healthy volunteers.
- herQLUS cohort: [27] ICU mechanically ventilated patients with visual assessment of pleural movement as the reference test.
- Lupo cohort: [22] Severe COVID-19 viral pneumonia patients undergoing PEEP titration with EIT as the reference test.

The Unito (short for “Università degli Studi di Torino”) cohort was retrieved from an ongoing study conducted at the University of Turin (IRB approval: 189582). The herQLUS and Lupo cohorts come from previously published trials conducted at the Amsterdam University Medical Centers (herQLUS, code for the “Quantitative Lung Ultrasound” trial, IRB approval: (2017_312#B201859) - Fig. 2S) [27] and at the Padua University Hospital (Lupo, “Lung Ultrasound, PEEP and Overdistension” trial, IRB approval: 0021712; Clinicaltrial.gov identifier: NCT04648657 - Fig. 2S) [22], respectively. Informed consent was previously obtained for the original studies in accordance with national regulations. A detailed description of the three cohorts is summarized in Table 1 and in Supplements.

**Fig. 1:**
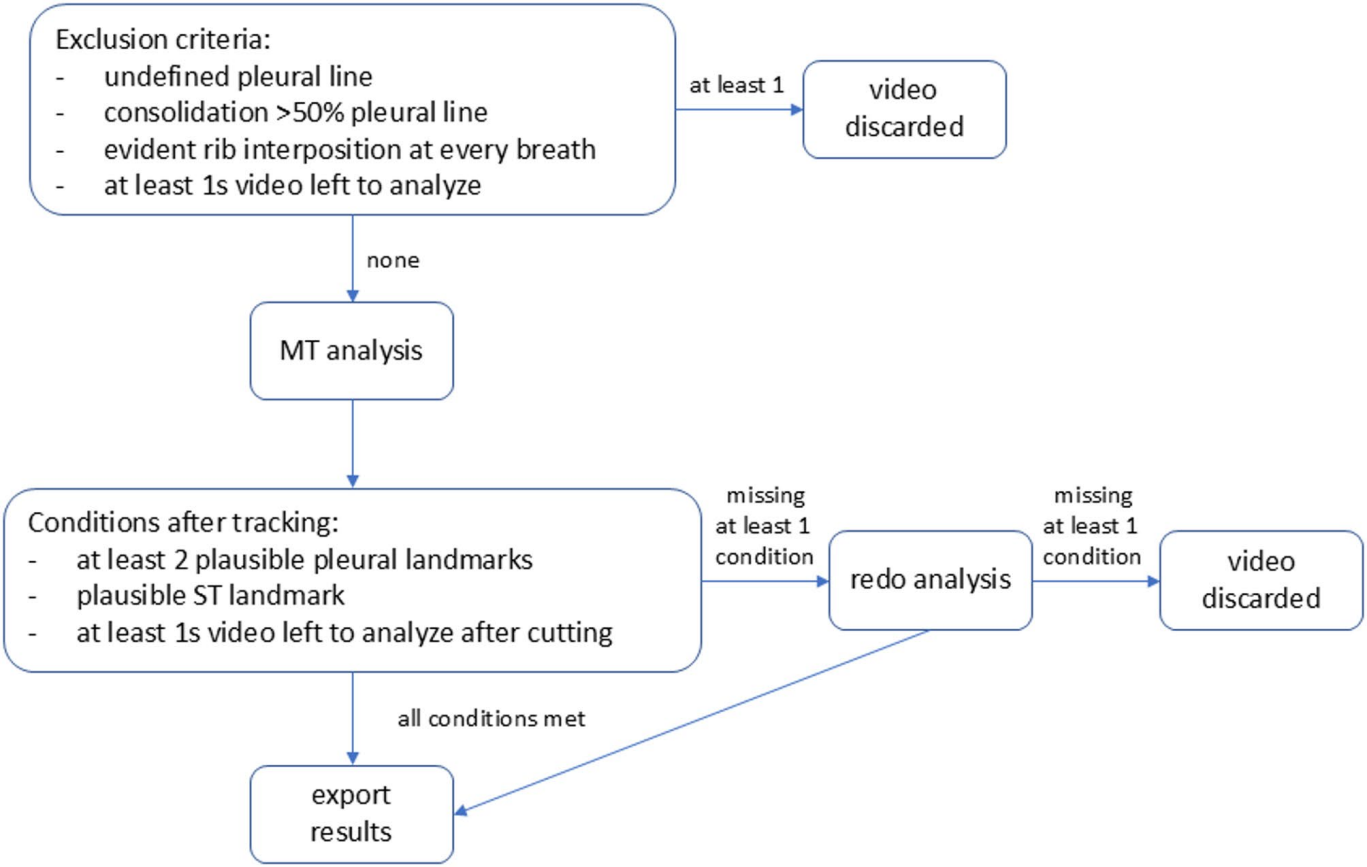
**Panel A**: motion tracking of the pleural line with three plausible pleural landmarks with soft tissue motion detected and affecting left and central landmarks Displacement/Time curve. To reduce noise, each motion–time curve was smoothed using a running median filter with a window of 5 data points, replacing each point with the median of its local neighborhood. The motion tracking data obtained on the transversal plane were normalized by redefining the zero point, ensuring that all landmarks started from a common reference position. Displacement/Time curves for each pleural landmark (central, right, and left) were normalized to account for probe movement, which was measured as soft tissue displacement in each LUS video clip. Specifically, if the motion-time curve of a given landmark showed a Pearson’s correlation ≥ 0.5 with the corresponding soft tissue motion-time curve, a new motion-time curve was generated for that specific landmark by subtracting the displacement value of the soft tissue landmark from the pleural landmark at each time point; **Panel B**: point-by-point correlation of left, central and right pleural landmarks with soft tissue position; **Panel C**: Displacement/Time curve of left, central, right and soft tissue landmarks before and after correlation and correction (see methods) when *R* > 0.5 (see methods). SP: motion tracking native starting point

**Table 1:** Overview of the three study cohorts

Patient characteristic	Unito		herQLUS		Lupo	
Videoclips (n)	176		336		358	
Format	.avi		.avi		.mp4	
Subjects (n)	7 (healthy)		40 (patients)		30 (patients)	
Fields	1 R-V-Cr 2 R-V-Ca 3 R-I-Cr 4 R-I-Ca 5 R-D-Cr 6 R-D-Ca	7 L-V-Cr 8 L-V-Ca 9 L-I-Cr 10 L-I-Ca 11 L-D-Cr 12 L-D-Ca	1 R-V-Cr 2 R-V-Ca 3 R-I-Cr 4 R-I-Ca 5 R-D-Cr 6 R-D-Ca	7 L-V-Cr 8 L-V-Ca 9 L-I-Cr 10 L-I-Ca 11 L-D-Cr 12 L-D-Ca	1 R-V-Cr 3 R-I-Cr	7 L-V-Cr 9 L-I-Cr
Probe	Linear (7–12 MHz)		Linear (7 MHz)		Linear (7–12 MHz)	
Ventilatory setting	SB		IMV – various modes		IMV – IPPV/VCV	
Conditions	*Breathing Maneuvers* FRC (*n* = 67) QB (*n* = 76) TLC (*n* = 21) RB (*n* = 12)		*Lung sliding (visual)* Absent (*n* = 61) Doubtful (*n* = 90) Moderate (*n* = 124) Extensive (*n* = 61)		*PEEP trial* Low PEEP (*n* = 119) Intermediate PEEP (*n* = 120) High PEEP (*n* = 119)	

R: right; L: left; V: ventral; I: intermediate; D: dorsal; Cr: cranial; Ca: caudal; IMV: invasive mechanical ventilation; SB: spontaneous breathing; IPPV: intermittent positive-pressure ventilation; VCV: volume-controlled ventilation; FRC: functional residual capacity; QB: quiet breathing; TLC: total lung capacity; RB: rapid breathing; PEEP: positive end-expiratory pressure

### LUS field definition

LUS videos from the entire study cohort were categorized using a twelve-field approach, where six regions for each hemithorax were identified based on anatomical landmarks (Fig. 1S). The LUS fields were further classified into ventral, intermediate, and dorsal regions based on their anatomical location:

**Fig. 2:**
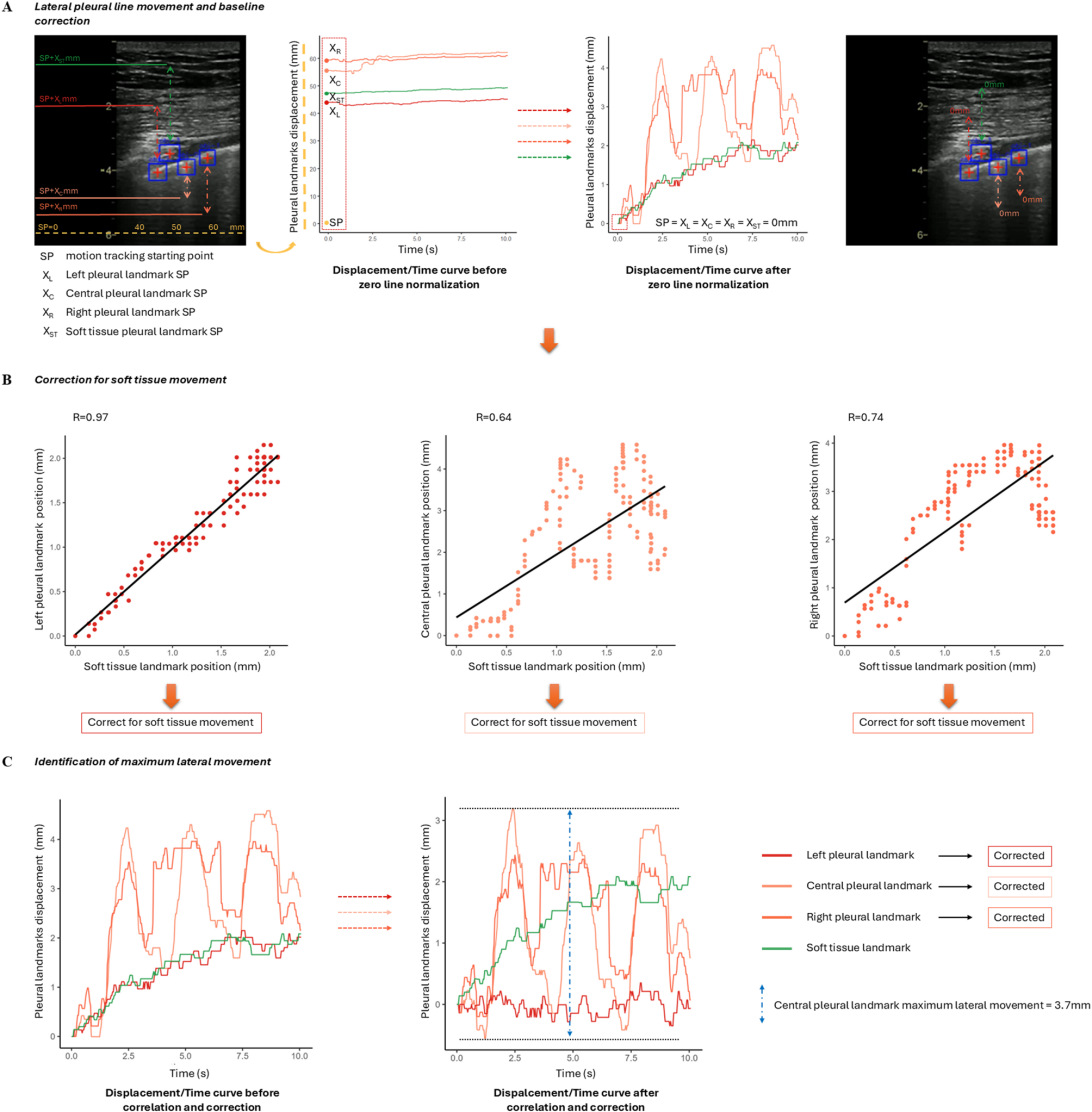
Flowchart illustrating the study design for motion technique analysis. LUS video clips were blindly reviewed from a qualitative perspective to determine their suitability for MT analysis of the pleural line. LUS video clips were discarded if any of the exclusion criteria were met. After motion tracking analysis, a post-hoc quality check was performed on the overlaying video file to ensure that all tracked landmarks followed a plausible trajectory. The following conditions had to be met: at least two pleural landmarks with a plausible tracking trajectory and a plausible ST landmark tracking trajectory and at least 1 s of video remaining for analysis after any necessary trimming. If at least one of the three conditions above was not met, the analysis was redone. If at least one condition was still unmet after the redo, the LUS video clip was discarded and not analyzed

- Ventral region: Fields 1, 2, 7, 8.
- Intermediate region: Fields 3, 4, 9, 10.
- Dorsal region: Fields 5, 6, 11, 12.

For the Lupo cohort, only fields 1, 3, 7 and 9 were available for analysis.

### Motion tracking of the pleural line

To track the motion of the PL, we used the standalone Windows^®^ version of an open-source MT application coded in Python, ‘Motion Tracker Beta’ [28]. The CSRT (Channel and Spatial Reliability Tracking) algorithm was used to track PL movement during the respiratory cycle [24, 28]. This tracker leverages discriminative correlation filters to robustly track “objects” (hereafter referred to as “landmarks”) across frames while maintaining accuracy even under challenging conditions such as occlusion, scale variations, and motion blur, all common challenges encountered in LUS video clips [24]. 

LUS video clips were blindly reviewed from a qualitative perspective to determine their suitability for MT analysis of the pleural line. LUS video clips were discarded if any of the exclusion criteria were met (Fig. 1).

Motion Tracker Beta allows tracking of multiple landmarks within the same video clip. MT of the PL was conducted as follows:

1. Defining the starting and ending time points for tracking.
2. Identifying three pleural landmarks (points) to track (one central, one on the right half, and one on the left half of the clip).
3. Choosing a soft tissue (ST) landmark to normalize for ST movement and probe translation-induced movements.
4. Calibrating the ruler.
5. Specifying the tracking algorithm in use (i.e. CSRT).
6. MT execution.

After motion tracking analysis, a post-hoc quality check was performed on the overlaying video file to ensure that all tracked landmarks followed a plausible trajectory (Fig. 1). If all conditions of quality control were met the video file with an overlay of the tracked landmarks and the results for each landmark (recording movement on a transversal and vertical plane) were exported into both a dedicated.mp4 and.csv file (Supplemental Video 1 to 5).

### Data importing and analysis

For this study, lateral motion was assessed using only the derived values from the horizontal transversal plane (Fig. 2, Panel A). Noise was reduced using a median running filter and ST motion was subtracted if most of the variation in PL movement was explained by movement of the soft tissue (Fig. 2, Panel B and C). ST displacement generated by cardiac activity was not removed due to the lack of a reproducible technical filter.

The maximum PL movement on the transversal plane was calculated for each pleural landmark as the greatest left-to-right excursion along the transversal plane, over the entire duration of the clip, of the point selected in the first frame. For each clip, the highest PL movement value among the three pleural landmarks was considered representative of that clip and this value was used to quantify PL movement.

### Endpoints

The PL movement was compared between breathing conditions in healthy volunteers and subjective quantification of lung sliding in ICU patients. Lung sliding was categorized into absent, doubtful, moderate, or extensive. PL movement was correlated to EIT-derived measures of overdistention, defined as the percentage of loss of compliance during a decremental PEEP trial.

### Statistical analysis

Data were tested for normal distribution using the Shapiro-Wilk test. Continuous variables are presented as mean ± standard deviation (SD) if normally or median and interquartile range if non-normally distributed. Categorical variables are presented as numbers and percentages. Comparisons were performed using paired or unpaired t-tests for parametric continuous variables as appropriate, the Wilcoxon signed-rank test and the Mann-Whitney U test or the Kruskal-Wallis test with Dunn’s post-hoc test with Benjamini-Hochberg adjustment for paired and unpaired non-parametric continuous variables, respectively. The Friedman test was applied to assess paired, non-parametric, continuous data measured at multiple time points. Categorical variables were analyzed with McNemar’s and Chi-square or Fisher’s exact test for paired and unpaired data, respectively.

A univariate ordered logistic regression was performed to examine the relationship between PL movement and sliding categories using the proportional odds model. Model performance was assessed using a confusion matrix, comparing predicted and actual sliding category classifications. A heatmap was used to visualize the results, with a color gradient indicating the percentage of predictions, where darker shades represented higher classification accuracy.

Spearman correlation analysis was first performed to assess the strength and direction of relationships between EIT derived lung aeration and PL movement or strain. Quadratic polynomial regression models were applied to each dataset to explore potential non-linear associations. Model comparisons were conducted using the Akaike Information Criterion (AIC), which accounts for both model fit and complexity to prevent overfitting. To assess the relative strength of competing models, the difference in AIC values (ΔAIC) was calculated as the difference between each model’s AIC and the lowest AIC observed. All statistical analyses were performed using R (version 4.3.2) within RStudio (version 2022.02.3, Build 492).

## Results

The Unito cohort consisted of seven healthy male subjects under 45 years of age with a Body Mass Index of less than 35 kg/m². Patient characteristics of the herQLUS and Lupo cohorts are detailed in Tables 1S and 2S. Of the 40 patients included in the herQLUS study and the 30 included in the Lupo study, 144 out of 480 and 2 out of 360 LUS fields, respectively, were not analyzed due to either the unavailability of LUS videos or the presence of exclusion criteria (Fig. 2S). Partial results were presented as a poster at the ATS 2025 International Conference in San Francisco [29].

### Motion tracking on healthy subjects

**Fig. 3:**
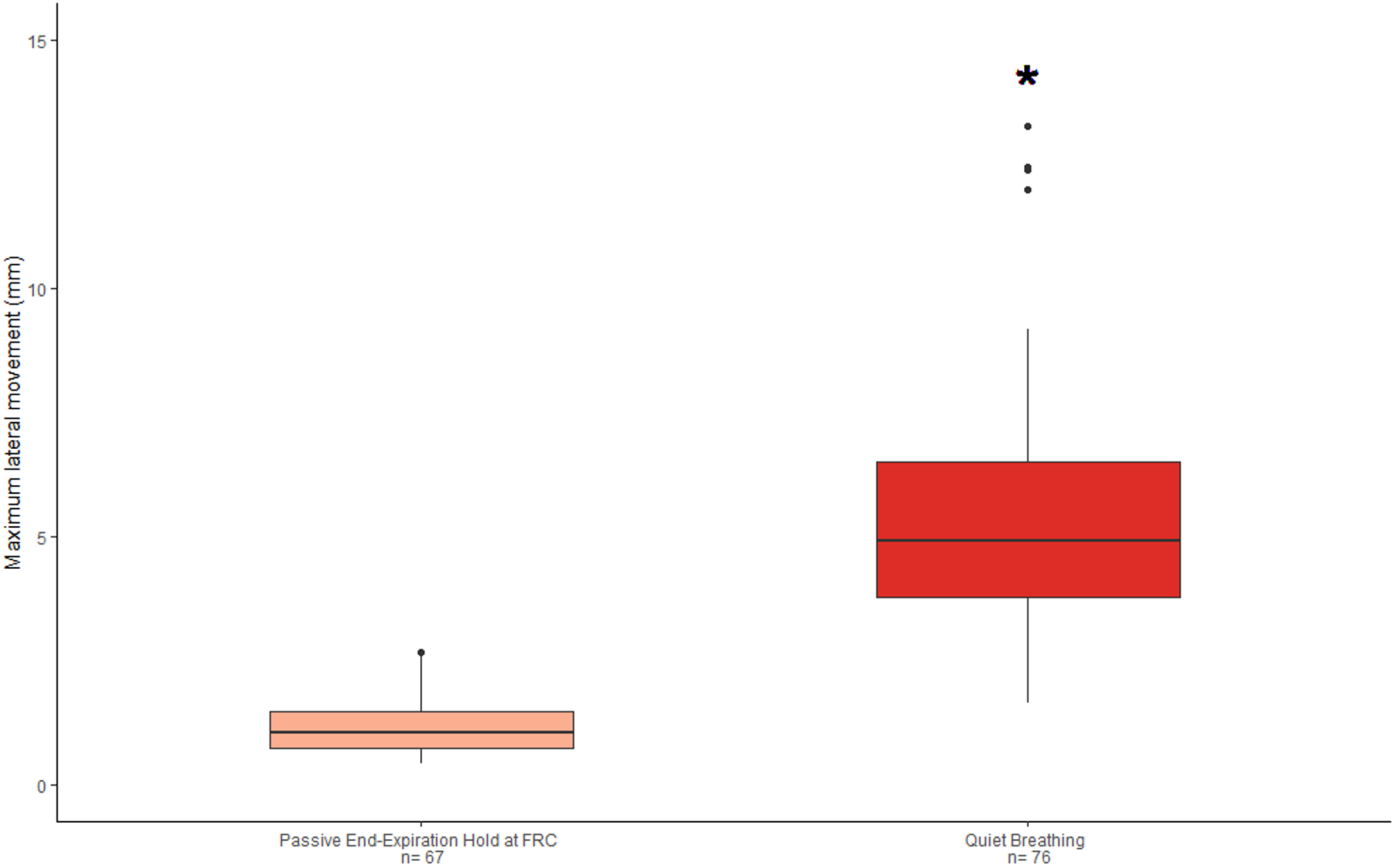
Boxplots showing pleural Maximum PL lateral movement in mm between ultrasound clips recorded at FRC and at QB. n: number of fields analyzed with motion tracking. *p < 0.01 vs. FRC category (Wilcoxon signed rank test with continuity correction for paired measurements). FRC: functional residual capacity; QB: quiet breathing; PL: pleural line

**Fig. 4:**
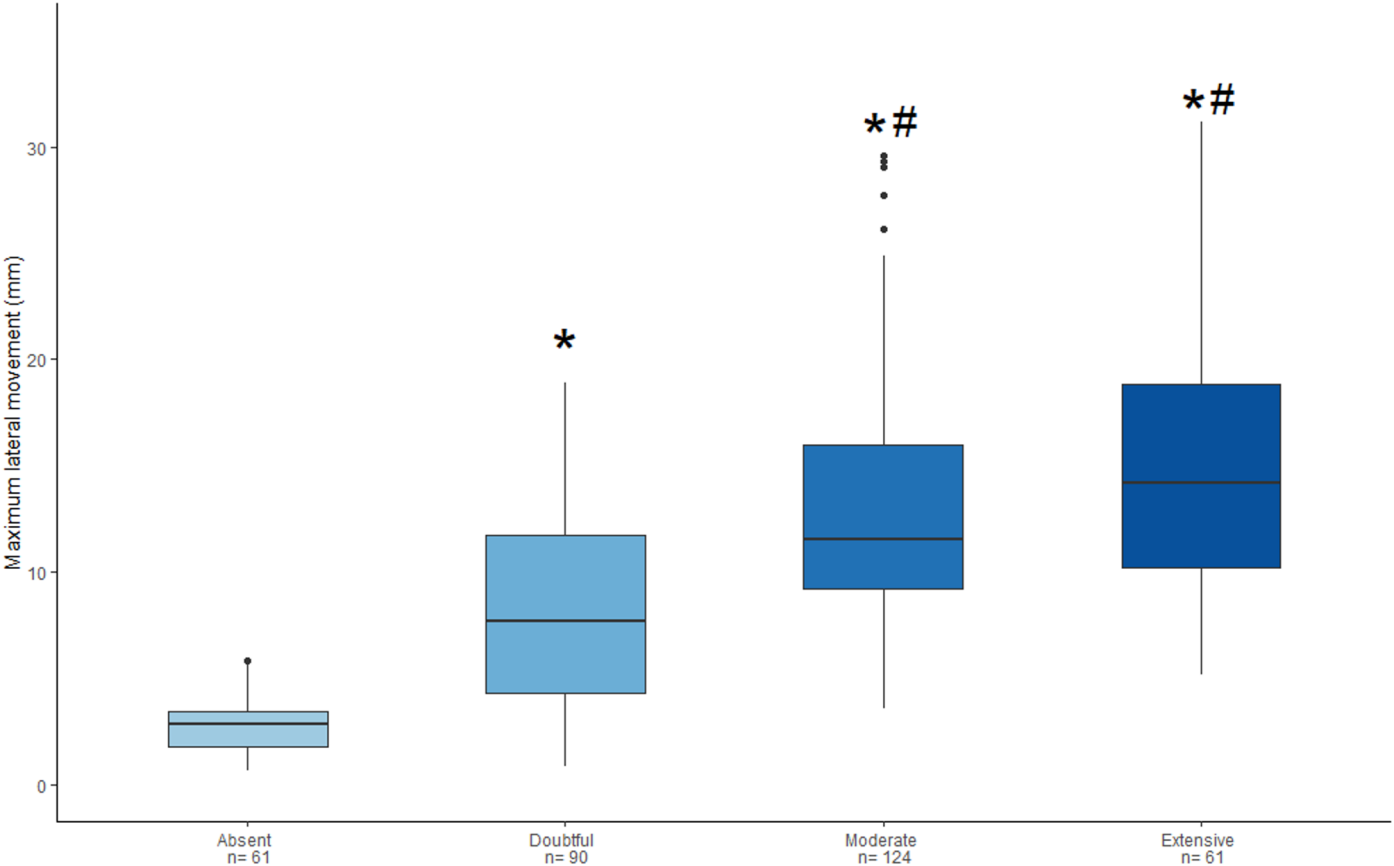
Boxplots showing maximum PL lateral movement in mm between sliding categories resulting from visual assessment. Absent: absence of sliding, Doubtful: doubt sliding, Moderate: evident not so wide sliding, Extensive: wide sliding. n: number of fields analyzed with motion tracking. *p < 0.01 vs. 0 category; #*p* < 0.01 vs. 1 category (Dunn test for multiple comparisons with Benjamini-Hochberg adjustment). PL: pleural line

**Fig. 5:**
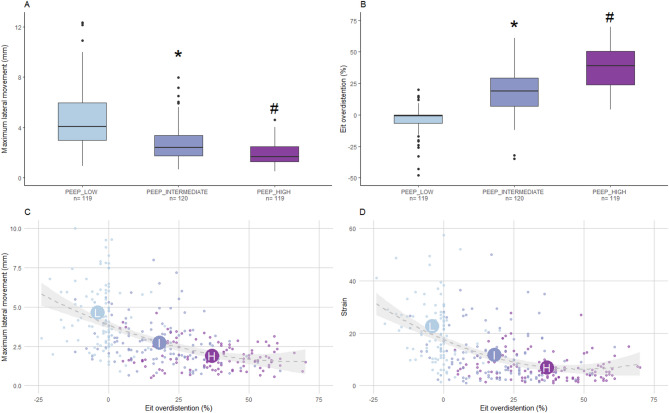
**Panel A**: Boxplots showing Maximum PL lateral movement in mm between PEEP categories. **Panel B**: Boxplots showing lung aeration in percentage of overdistention or atelectasis between PEEP categories. LOW: the lowest PEEP available for each subject; INTERMEDIATE: PEEP 14 cmH2O; HIGH: the highest PEEP available for each subject. n: number of fields analyzed with motion tracking. *p < 0.01 vs. LOW category; #*p* < 0.01 vs. INTERMEDIATE category (Friedman test with Dunn’s post-hoc analysis with Bonferroni correction). **Panel C**: Scatter plot illustrating the relationship between lung aeration and maximum lateral movement across different PEEP levels (LOW, INTERMEDIATE, HIGH). **Panel D**: Scatter plot illustrating the relationship between lung aeration and strain measured with speckle tracking algorithm across different PEEP levels (LOW, INTERMEDIATE, HIGH). Polynomial quadratic regression lines are fitted to the data, indicating trends within each group. Centroids of the means for each PEEP level are labeled (L, I, H) and reflect average values of maximum lateral movement and lung aeration. PL: pleural line; PEEP: positive end-expiratory pressure

Median overall PL movement during a passive end-expiration hold at Funcutonal Residual Capacity (FRC) was lower compared to Quiet Breathing (QB) (*p* < 0.0001, Fig. 3). Data from a subset of subjects showed that PL movement during a maximal inspiration hold at Total Lung Capacity (TLC) was not different from PL movement during a passive end-expiration hold at FRC (*p* = 0.9563; Fig. 3S). Similarly, PL movement during Rapid Breathing (RB) did not differ from PL movement during QB (*p* = 0.266; Fig. 3S). PL movement variation across ventilation patterns (FRC, TLC, QB and RB) stratified for regional fields is shown in Fig. 4S. PL movement at FRC and TLC was lower than at QB and RB irrespective of anatomical locations (*p* < 0.001; Fig. 5S). PL movement was heterogeneous between individuals, but similar trends were identified between breathing patterns, with lower PL movement at FRC than with QB (Fig. 5S).

### Qualitative sliding assessment

In the herQLUS cohort, median PL movement was lower in clips without lung sliding compared to the other groups, and in clips with doubtful sliding compared to moderate and extensive sliding (*p* < 0.0001). However, PL movement was not different between clips classified as having moderate or extensive sliding (*p* = 0.0761; Fig. 4). Figure 6S displays the PL movement across sliding categories stratified for the regional fields, showing no significant differences across anatomical locations.

Ordinal logistic regression analysis revealed a significant positive relationship between PL movement and the likelihood of higher sliding categories. Specifically, for each 1 mm increase in PL movement, the odds of progressing to a higher sliding category increased by approximately 25%.

Figure 7S displays the model’s performance in a confusion matrix. Accuracy was adequate for no sliding and doubtful sliding categories, but lower for the moderate and extensive sliding groups. Taken together, PL movement can identify clips with absent to doubtful sliding, which could represent overdistention.

### Strain and overdistension

The EIT-guided decremental PEEP trial performed in the LUPO cohort [22] resulted in overdistention detected by EIT proportional to the applied PEEP levels. Separating the applied PEEP levels into three groups, the median PL movement was significantly higher in the *low* than in both the *intermediate* (*p* < 0.0001) and in the *high* PEEP group (*p* < 0.0001, Figs. 5 and 10S). Similarly, median overall EIT-derived overdistention percentage (OD%) was significantly lower in the *low* than in both the *intermediate* (*p* < 0.0001) and in the *high* PEEP group (*p* < 0.0001, Fig. 5). Median PL movement and EIT overdistention % values at different PEEP levels stratified per for ventral and intermediate fields are reported in the supplements (Figs. 8S, 9S and 10S).

PL movement and strain showed a significant inverse correlation with EIT overdistention % (ρ=−0.593, *p* < 0.0001; ρ =−0.563, *p* < 0.0001, respectively). Polynomial regression analysis was performed to evaluate the ability of PL movement and strain to predict EIT overdistention % (Fig. 5). The second-degree polynomial model for PL movement explained 31.4% of the variance in EIT overdistention % (R²=0.314, *p* < 0.0001). Both the first-order (β = −12.53, *p* < 0.0001) and second-order (β = 0.73, *p* < 0.0001) terms were statistically significant, indicating a non-linear relationship. Similarly, the polynomial model for strain accounted for 30.0% of the variance in EIT overdistention % (R²=0.300, *p* < 0.0001). Both the first-order (β=−1.70, *p* < 0.0001) and second-order (β = 0.015, *p* < 0.0001) terms were also significant, suggesting also a non-linear association. Polynomial regression analysis, restricted to the ventral and intermediate fields, is shown in the supplements (Fig. 8S S, respectively). After fitting second-degree polynomial regression models on a common dataset (excluding missing values), the model using PL movement as predictor explained slightly more variance in EIT overdistention % (adjusted R²=0.306) than the model based on strain (adjusted R²=0.296). Additionally, the PL movement model yielded a lower AIC (2974.2 vs. 2979.0 - ∆AIC = 4.8), indicating that PL movement is a stronger predictor of EIT overdistention % than strain.

## Discussion

The main finding of this study is that PL motion tracking is feasible using a simplified approach that leverages a widely available and easily applicable open-source algorithm. This allows the user to detect PL movements in common video files without specific requirements. Moreover, PL motion tracking accurately detected the presence or absence of sliding in both healthy volunteers during spontaneous breathing and in mechanically ventilated patients. Additionally, PL movement outperformed strain analysis in detecting overdistention.

Our study is the first to apply this technique extensively in both healthy subjects and mechanically ventilated patients [18]. Speckle-tracking derived strain analysis of the PL has previously shown to be feasible in both mechanically ventilated patients [17, 22] and healthy subjects during spontaneous breathing [16]. Initial clinical applications focused on the detection of pneumothorax [19–21], providing no further characterization of lung sliding beyond assessing its presence or absence. Pleural MT has been evaluated in mechanically ventilated patients by measuring the amplitude of lateral displacement of B-lines [30]. However, this approach relied on manual tracking and cannot be applied when B-lines are absent. In contrast, our method remains effective regardless of their presence. More recently, tissue displacement during the respiratory cycle was described in a proof-of-concept paper using a custom-made approach [25]. Although promising, this approach was tested in two subjects only and validated using simulated lung motion images. Speckle-tracking has also been applied to derive strain during an incremental PEEP trial with EIT as reference standard, to assess overdistention [22]. Our results are comparable to those obtained with strain analysis and seem equally accurate in detecting changes in overdistention. Recently, Tonelotto et al. proposed a simple method to detect lung overdistention in mechanically ventilated patients by counting A-lines in normally aerated regions [31]. However, this technique may be inaccurate when the presence of B-lines prevents visualization and counting of A-lines in overdistended areas. Motion tracking of the PL can be applied regardless of lung aeration and is not limited to the detection of overdistension, as it can directly assess and quantify pleural sliding. Nevertheless, it may be suboptimal in distinguishing a reduction in sliding caused by lung overinflation from that caused by lobar consolidation. In these cases, pleural motion is not informative, as the consolidation itself carries clear diagnostic meaning. Thus, pleural motion should be regarded as complementary to the LUS score, providing additional quantification and refinement. Despite this, PL movement demonstrated good accuracy in detecting overdistension in the Lupo cohort, in which nearly all patients exhibited a non-focal pattern, which represents the theoretically more challenging scenario for training and validating the motion tracking technique. More generally, the approach here described showed greater flexibility compared to previous studies in terms of software availability and customization, as well as in its ability to work with clips of variable resolution and format independent of Digital Imaging and Communications in Medicine (DICOM) image acquisition.

In our cohorts, motion tracking of the PL was significantly different from zero, even in healthy volunteers, holding their breath at FRC or TLC. This might be explained by the presence of lung pulse, as well as by the incomplete normalization of pleural landmarks with ST movement, considering the cutoffs used for correlations. Similar results were found during breath hold by Fung et al. [25] Additionally, we chose to use the maximum lateral displacement to identify PL movement, which minimizes false negatives but increases the risk of false positives. Individual absolute PL movement values were found to be relatively higher in the herQLUS cohort compared to the Lupo cohort. This difference could be explained because of the different ventilatory settings applied in the two cohorts (Table 1S and 2 S). Regardless of these differences, the key finding is the ability of motion tracking to capture pleural motion variations within individual subjects, as demonstrated by analyses conducted in both the healthy subjects (Fig. 5S) and Severe COVID-19 viral pneumonia patients (Fig. 10S).

Strengths of our study include the implementation of a novel approach using open-source, highly versatile software with unlimited theoretical applications, free from format or resolution constraints. The software was not specifically designed to track the PL; however, the code was written to be versatile and applicable to a wide range of biomedical and engineering applications. Moreover, the code is completely open-source, allowing the software to be adapted to any circumstance, as needed. Furthermore, we demonstrated consistency by obtaining the same signal across three different datasets, collected at different times and centers, using different machines and reference tests. In addition, our software allows to obtain raw data of point-to-point movement along the X and Y axes, frame by frame. This enables to have full control over the data acquisition and analysis process. Moreover, the method described showed broad applicability across videos from diverse cohorts. This demonstrates its robustness and generalizability, suggesting it could be widely implemented in clinical or research settings rather than being restricted to a specific subset of videos and/or formats. Lastly, our findings suggest that PL movement is at least as good, and possibly better, than strain analysis in predicting overdistention as measured with EIT.

This study has some limitations. First, vertical movement was not integrated into the calculation, which may have introduced some bias when the PL was not perfectly aligned with the horizontal plane. The decision to focus solely on the transversal plane was intentional, aimed at understanding and explaining lateral movement without adding further complexity. However, the software can analyze both X- and Y-axis movements of a structure over time. Future analyses could incorporate a more comprehensive 2D approach, such as Euclidean displacement analysis, as well as evaluations of velocity and acceleration of PL movement. Second, a method for filtering out lung pulse was not applied. However, this is expected to introduce only a minor bias by adding some movement to the PL without significantly affecting the analysis. Future analytical improvements could focus on removing this signal entirely, even though this would likely require ECG tracing. Third, the operator performing motion tracking was, per protocol, unblinded to the clip. This was necessary due to the pioneering nature of this study, where understanding the behavior of the tracking algorithm during individual clip analysis was essential. Future analyses will aim to develop a more precise method for normalizing pleural landmarks relative to ST movement. Fourth, motion tracking was performed on lung ultrasound clips of varying duration, and standardization of the respiratory cycle during the analysis was not possible.

Motion tracking of the PL may represent an easy-to-implement tool for post-hoc analysis of LUS video clips. It could theoretically be integrated into the software of any ultrasound machine, allowing clinicians to perform analysis in a telemedicine context. Additionally, it could complement LUS score evaluation, particularly when an A-pattern is used to differentiate between normal aeration and overinflation. Moreover, it could be used to detect overdistention during titration of PEEP and tailoring mechanical ventilation, thus further mitigating the risk of VILI. Another strength of this approach lies in the motion tracking of ST, which can provide accurate PL motion values not only in research-based recorded clips but also in real-world scenarios, where probe translation and movement artifacts may be present and could interfere with strain evaluation using other software.

## Conclusions

Motion tracking of the PL was feasible and accurately detected and quantified lung sliding and assessed overdistention during mechanical ventilation. This was achieved using freely available open-source software, which is relatively easy to use, and capable of accurately detecting PL movements in common video files. Further prospective studies specifically designed to apply this approach and to improve it with automated PL recognition and analysis are needed to widely determine its usefulness in clinical applications.

## Supplementary Information

Supplementary Material 1

Supplementary Material 2

Supplementary Material 3

Supplementary Material 4

Supplementary Material 5

Supplementary Material 6

## Data Availability

The datasets generated and/or analysed during the current study are not publicly available due to privacy and ethical regulations but are available from the corresponding author on reasonable request.

## Abbreviations

PL: Pleural line
ARDS: Acute respiratory distress syndrome
ICU: Intensive care unit
EIT: Electrical impedance tomography
LUS: Lung Ultrasound
MT: Motion tracking
OpenCV: Open source computer vision library
CSRT: Channel and spatial reliability tracking
ST: Soft tissue
PEEP: Positive end-expiratory pressure
AIC: Akaike information criterion
FRC: Functional residual capacity
QB: Quiet breathing
TLC: Total lung capacity
RB: Rapid breathing
DICOM: Digital imaging and communications in medicine
VILI: Ventilator-induced lung injury
