# Supplementary material for: Tracking pleural sliding motion to assess lung overdistention using an open source algorithm: a proof-of-concept study on lung ultrasound scans
Source: Crit Care. 2026 Jan 22;30:37. doi: 10.1186/s13054-025-05742-8 (PMC12829235; doi:10.1186/s13054-025-05742-8)
Supplement: Supplementary file 1 — Supplementary Material 1 [file 13054_2025_5742_MOESM1_ESM.pdf]

Supplemental digital content

Title:

Tracking Pleural Sliding Motion to Assess Lung Overdistention using an Open Source Algorithm: A Proof-of-Concept Study on Lung Ultrasound Scans

Running title:

Lung Ultrasound Motion Tracking of the Pleural Line

Authors:

Costamagna, A.[1,2] MD; Smit, M.R.[2] PhD; Pivetta, E.E.[3] MD, PhD; Persona, P.[4], MD, PhD; Navalesi, P.[5], MD, FERS; Pisani, L.[6], MD, PhD; Schultz, M.J.[2,7] MD, PhD; Brazzi, L[1], MD, PhD; Fanelli, V.[1], MD, PhD; Tuinman, P.R.[2], MD PhD; Bos, LDJ[2] MD, PhD

Table of contents

- Material and Methods
- Results
- Table 1S and 2S
- Figure legends
- Figures from Figure 1S to Figure 10S

Materials and methods

Study population

Unito cohort

The Unito cohort consists of 176 LUS videos collected from an ongoing study aimed at evaluating the performance of some in-training LUS operators in obtaining LUS videos clips. For this analysis, only the videos obtained from healthy subjects were included. Among all the available LUS videos, four conditions were identified, when present in the same study subjects:

- FRC: video clips recorded during a passive end-expiration hold at functional residual capacity (n = 67)
- QB: video clips recorded during quiet breathing with tidal volume respiration (n = 76)
- TLC: video clips recorded during a maximal inspiration hold at total lung capacity (n = 21)
- RB: video clips recorded during rapid breathing at a respiratory rate (RR) > 25 bpm (n = 12)

herQLUS cohort

The herQLUS cohort is composed of a total of 336 LUS video clips coming from 40 patients enrolled in a previously published study. For a detailed description of the methods, please refer to the main paper. Briefly, invasively mechanically ventilated patients with a clinical indication for chest CT underwent a 12-region LUS examination. Six areas on each hemithorax (two

ventral, two lateral, and two posterolateral regions) were scanned using a high-frequency linear probe with a transverse approach. LUS video clips were blindly assessed by an experienced LUS researcher (AC) and categorized into four groups based on visual assessment of pleural movement:

Absent: total absence of lung sliding (n=61)

Doubtful: Doubtful presence of lung sliding (n=90)

Moderate: Evident lung sliding, but of limited extension (n=124)

Extensive: Evident and extensive lung sliding (n=61)

Lupo cohort

The Lupo cohort is composed of 358 LUS video clips coming from 30 patients enrolled in a previously published trial. For a detailed description of the methods, please refer to the main paper. Briefly, all consecutive adult patients intubated for acute hypoxemic respiratory failure (PaO2/FiO2 < 300 mmHg) were enrolled. An EIT-guided decremental PEEP trial (from 20 cmH_2O to 2 cmH_2O below the PEEP level corresponding to the highest quasi-static compliance of the respiratory system) was conducted to evaluate PEEP-induced overdistension. The decremental PEEP trial was analyzed using the software (Pulmovista, Draeger, Lübeck, Germany), and the amount of regional compliance decrease, resulting from both lung collapse and overdistention, was calculated for each PEEP step according to Costa et al. [1]. Briefly, the best compliance of each pixel is determined, corresponding to the PEEP level at which maximal tidal impedance variation is recorded. For each PEEP level, a decrease in pixel compliance is interpreted as lung collapse or overdistention when it occurs at lower or higher PEEP than the PEEP level associated with the pixel's best compliance, respectively. Regions of interest were defined for each EIT image: the thorax was divided into four equally spaced layers in the

ventral-to-dorsal direction, with each layer further divided into two symmetrical halves by the vertical line at the center of the image. For each region of interest, the software calculates the average reduction in compliance due to overdistention (OD%). As lung ultrasound was performed in the anterior part of the thorax, only data from the right and left most anterior regions were considered. (**Figure 1S**)

LUS was performed during each PEEP step using a linear probe (7–12 MHz) in four regions, positioned either 1 cm above or below the EIT belt and aligned parallel to it within the intercostal space on both the right and left sides of the patient. EIT overdistention percentage (OD%) values were defined as the percentage of loss of compliance during a decremental PEEP trial. Longitudinal speckle tracking was applied to each LUS, and peak strain values were recorded (GE EchoPAC version 203 (GE HealthCare, USA)). Three LUS video clips have been analyzed in this study for each patient, corresponding to three PEEP categories:

- LOW: the lowest PEEP available for each subject (n=119)
- INTERMEDIATE: PEEP=14cmH2O (n=120)
- HIGH: the highest PEEP available for each subject (n=119)

Results

Qualitative sliding assessment

Median overall PL movement was 2.8 mm (IQR 1.8 – 3.4 mm) in the absence of lung sliding, 7.7 mm (IQR 4.3 – 11.7 mm) with doubtful lung sliding (group 1), 11.6 mm (IQR 9.2 – 15.9 mm) with moderate, and 14.2 mm (IQR 10.2 – 18.8 mm) with evident lung sliding. PL movement was significantly lower in the Absent group compared to the other categories, and in Doubtful compared to Moderate and Extensive groups ($p < 0.0001$). However, PL movement did not differ significantly between Moderate and Extensive groups ($p = 0.0761$; **Figure 4**). Violin dispersion plots showing PL movement in millimeters across sliding categories are presented overall and separately for the ventral, intermediate, and dorsal fields (**Figure 6S**, panel A and B, respectively).

The ordinal logistic regression analysis revealed a significant positive relationship between PL movement and the likelihood of higher sliding categories. Specifically, for each 1 mm increase in PL movement, the odds of progressing to a higher sliding category increased by approximately 25%. The coefficient for PL movement was 0.2251 (Standard Error = 0.02046), and the t-value was 11.00, with a highly significant p-value of 3.66×10^{-28}, indicating a very strong association. The intercepts, which define the thresholds between the categories, were as follows: the threshold for moving from Absent to Doubtful category was 0.2439 (Standard Error = 0.2033, p = 0.23). The threshold for moving from Doubtful to Moderate category was 2.0943 (Standard Error = 0.2357, p = 6.31 × 10. Finally, the threshold for moving from Moderate to Extensive category was 4.4301 (Standard Error = 0.3217, p = 3.83 × 10. PL movement is strongly associated with increased levels of sliding, with particularly strong effects seen between Doubtful and Moderate categories and between Moderate and Extensive categories.

The model showed a residual deviance of 742.30 and an AIC of 750.30, indicating a good fit to the data (**Figure 7S, Panel A**).

The model's performance was further assessed using a confusion matrix (**Figure 7S, Panel B**). The overall accuracy of the model was 48.51%, with a 95% confidence interval ranging from 43.05% to 54%. The No Information Rate (NIR), representing the accuracy of predicting the most frequent class, was 36.9%, and the p-value for the accuracy being greater than NIR was highly significant ($p = 9.096 \times 10^{-6}$). The Kappa statistic, which quantifies the agreement between predicted and observed values, was 0.2714, indicating a moderate level of agreement. Performance metrics, including sensitivity, specificity, and predictive values, varied across the different sliding categories. Sensitivity was highest for the Moderate (0.6371) and lowest for the Extensive category (0.2623), suggesting that the model was most effective at detecting sliding in Moderate sliding group. Specificity was highest for the Extensive group (0.9236), indicating excellent performance in correctly predicting no sliding for this category, and lowest for the Doubtful group (0.7317). Positive predictive value (PPV) and negative predictive value (NPV) also exhibited variation across classes, with absence of sliding having the highest PPV (0.6905) and NPV (0.8912).

Table 1S. Patient characteristics of the herQLUS cohort

**Patient characteristic**	**N=40**
Age (years)	59 (50-69)
Female N (%)	11 (28)
BMI (Kg/m^2)	25 (22-32)
Apache II	20 (15-24)
ICU mortality N (%)	14 (35)
Reason for ICU admission N (%)	
Planned surgery	5 (13)
Emergency surgery	5 (13)
Trauma	2 (5)
Medical condition	26 (65)
Other	2 (5)
Reason for intubation N (%)	
Cardiac arrest	3 (8)
Planned postoperative MV	7 (18)
Depressed level of consciousness	3 (8)
Acute Respiratory Failure	25 (63)
Other	2 (5)
ARDS N (%)	28 (70)
Bilateral opacities N (%)	29 (73)
Cardiac overload N (%)	8 (20)
Ventilatory mode N (%)	
PCV	14 (35)
PSV	24 (60)
ASV	2 (5)
TV (mL)	452 (390-555)
PEEP (cmH_2O)	8 (5-10)
RR (bpm)	24 (18-32)
P/F ratio (mmHg)	204 (134-271)

List of abbreviations: BMI: Body Mass Index; ICU: Intensive Care Unit; COPD: Chronic Obstructive Pulmonary Disease; ARDS: Acute Respiratory Distress Syndrome; MV: Mechanical Ventilation; PCV: Pressure-Controlled Ventilation; PSV: Pressure-Support Ventilation; ASV: Adaptive Support Ventilation; TV: Tidal Volume; PEEP: Positive End-Expiratory Pressure; RR Respiratory Rate; P/F: Arterial Partial Pressure of Oxygen to Fraction of Inspired Oxygen. Data are expressed as median (IQR) for continuous and as frequency and percentage for categorical variables.

Table 2S. Patient characteristics of the Lupo cohort

**Patient characteristic**	**N=30**
Age (years)	68 (70-74)
Female N (%)	9 (23)
BMI (Kg/m^2)	28 (26-34)
Covid-19 diagnosis N(%)	28 (70)
Reason for ICU admission N (%)	
COVID-19	30 (100)
Cause of respiratory failure N (%)	
Severe COVID-19 Pneumonia	30 (100)
Reason for intubation N (%)	
Acute Respiratory Failure	30 (100)
Cardiac overload N (%)	0 (0)
Ventilatory mode N (%)	
IPPV-VCV	30 (100)
TV (mL)	400 (380-420)
PEEP (cmH_2O)	Titrated by protocol

List of abbreviations: BMI: Body Mass Index; ICU: Intensive Care Unit; IPPV-VCV: Intermittent Positive Pressure Ventilation-Volume Controlled Ventilation; TV: Tidal Volume; PEEP: Positive End-Expiratory Pressure. Data are expressed as median (IQR) for continuous and as frequency and percentage for categorical variables.

Figure legend

Figure 1S. Panel A and B: exemplary photo and graphical representation of the 12 LUS fields: fields 1 and 7 at the 2nd-3rd intercostal (IC) spaces along the midclavicular line; fields 2 and 8 at the 5th-6th IC spaces along the midclavicular line; fields 3 and 9 at the 3rd-4th IC spaces along the anterior axillary line; fields 4 and 10 at the 6th-7th IC spaces along the anterior axillary line; fields 5 and 11 at the 4th-5th IC spaces along the posterior axillary line; fields 6 and 12 at the 7th-8th IC spaces along the posterior axillary line and over the diaphragm. © Marry R. Smit et al. Creative Commons CC-BY. [27] Panel C and D: exemplary photos of an ARDS patient's right and left hemithorax equipped with an EIT belt, showing the region examined for assessing pleural motion. For study purposes, the belt was strapped slightly superior to the level indicated in the figure. Pictures courtesy of Dräger.

Figure 2S. Study flowchart for herQLUS and Lupo cohorts

Figure 3S. Scatter plot illustrating the maximum lateral movement in mm for FRC and TLC conditions (Panel A) and for QB and RB conditions (Panel B). Each point represents an individual observation, with lines connecting paired observations coming from the same subject. FRC: functional residual capacity; TLC total lung capacity; QB: quiet breathing; RB: rapid breathing.

Figure 4S. Violin dispersion plots showing Maximum lateral movement in mm between healthy subjects at FRC, TLC, QB and RB, overall (Panel A) and in the ventral, intermediate and dorsal fields (Panel B). The horizontal lines within each violin correspond to the 25th, 50th (median), and 75th percentiles. FRC: functional residual capacity; TLC total lung capacity; QB: quiet

breathing; RB: rapid breathing. *p<0.01 vs FRC; #p<0.01 vs TLC, Kruskal–Wallis test followed by Dunn's post hoc test

Figure 5S. Scatter plot illustrating the maximum lateral movement in mm for FRC and QB conditions, overall (Panel A) and in the ventral, intermediate and dorsal fields (Panel B, C and D, respectively). Each point represents an individual observation, with lines connecting paired observations coming from the same subject. FRC: functional residual capacity; QB: quiet breathing. *p<0.01 vs FRC, Wilcoxon Signed-Rank test

Figure 6S. Violin dispersion plots showing maximum lateral movement in mm between sliding categories resulting from visual assessment overall (Panel A) and in the ventral, intermediate and dorsal fields (Panel B). The horizontal lines within each violin correspond to the 25th, 50th (median), and 75th percentiles. Absent=absence of sliding, Doubtful=doubt sliding, Moderate=evident not so wide sliding, Extensive=wide sliding. *p<0.01 vs Absent; #p<0.01 vs Doubtful; °p<0.05 vs ventral, Kruskal–Wallis test followed by Dunn's post hoc test

Figure 7S. Panel A: univariate ordered logistic regression curves showing the predicted probabilities of sliding categories resulting from visual assessment of lung sliding (Absent, Doubtful, Moderate and Extensive, from light to dark blue, respectively) as a function of maximum lateral movement. The lines represent the estimated probabilities for each sliding category resulting from visual assessment of lung sliding, based on the univariate ordered logistic regression model. Panel B: Confusion matrix displaying the predicted versus actual classifications of sliding categories based on the ordered logistic regression model using maximum lateral movement. Each cell shows the frequency of observations and the corresponding percentage of predictions for each actual sliding category (Actual) compared to

the predicted categories (Predicted). The color gradient represents the percentage of predictions, with lighter shades indicating lower percentages and darker shades indicating higher percentages.

Figure 8S. Ventral fields. Panel A: Boxplots showing Maximum lateral movement in mm between PEEP categories. Panel B: Boxplots showing lung aeration in percentage of overdistention or atelectasis between PEEP categories. LOW: the lowest PEEP available for each subject; INTERMEDIATE: PEEP 14 cmH2O; HIGH: the highest PEEP available for each subject. n=number of fields analyzed with motion tracking. *p<0.01 vs LOW category; #p<0.01 vs INTERMEDIATE category (Friedman test with Dunn's post-hoc analysis with Bonferroni correction). Panel C: Scatter plot illustrating the relationship between lung aeration and maximum lateral movement across different PEEP levels (LOW, INTERMEDIATE, HIGH). Panel D: Scatter plot illustrating the relationship between lung aeration and strain measured with speckle tracking algorithm across different PEEP levels (LOW, INTERMEDIATE, HIGH). Polynomial quadratic regression lines are fitted to the data, indicating trends within each group. Centroids of the means for each PEEP level are labeled (L, I, H) and reflect average values of maximum lateral movement and lung aeration.

Figure 9S. Intermediate fields. Panel A: Boxplots showing Maximum lateral movement in mm between PEEP categories. Panel B: Boxplots showing lung aeration in percentage of overdistention or atelectasis between PEEP categories. LOW: the lowest PEEP available for each subject; INTERMEDIATE: PEEP 14 cmH2O; HIGH: the highest PEEP available for each subject. n=number of fields analyzed with motion tracking. *p<0.01 vs LOW category; #p<0.01 vs INTERMEDIATE category (Friedman test with Dunn's post-hoc analysis with Bonferroni correction). Panel C: Scatter plot illustrating the relationship between lung aeration

and maximum lateral movement across different PEEP levels (LOW, INTERMEDIATE, HIGH). Panel D: Scatter plot illustrating the relationship between lung aeration and strain measured with speckle tracking algorithm across different PEEP levels (LOW, INTERMEDIATE, HIGH). Polynomial quadratic regression lines are fitted to the data, indicating trends within each group. Centroids of the means for each PEEP level are labeled (L, I, H) and reflect average values of maximum lateral movement and lung aeration.

Figure 10S. Scatter plot illustrating the maximum lateral movement in mm between PEEP categories, overall (Panel A) and in the ventral right, intermediate right, ventral left and intermediate left fields (Panel B, C, D and E, respectively). Each point represents an individual observation, with lines connecting paired observations coming from the same subject. LOW: the lowest PEEP available for each subject; INTERMEDIATE: PEEP 14 cmH2O; HIGH: the highest PEEP available for each subject.

Video 1. Example video of the pleural line motion tracking in a healthy subject during quiet breathing

Video 2. Example video of the pleural line motion tracking in a healthy subject during breath hold at end-expiration (FRC)

Video 3. Example video of pleural line motion tracking in a patient with severe COVID-19 viral pneumonia and absent sliding, potentially due to overdistension during mechanical ventilation

Video 4. Example video of pleural line motion tracking in a patient with severe COVID-19 viral pneumonia and extensive sliding, during mechanical ventilation

Video 5. Example video of pleural line motion tracking in a patient with severe COVID-19 viral pneumonia and absent sliding, showing artifact due to soft tissue movement from probe translation, correctly followed by the tracking algorithm

Figure 1S

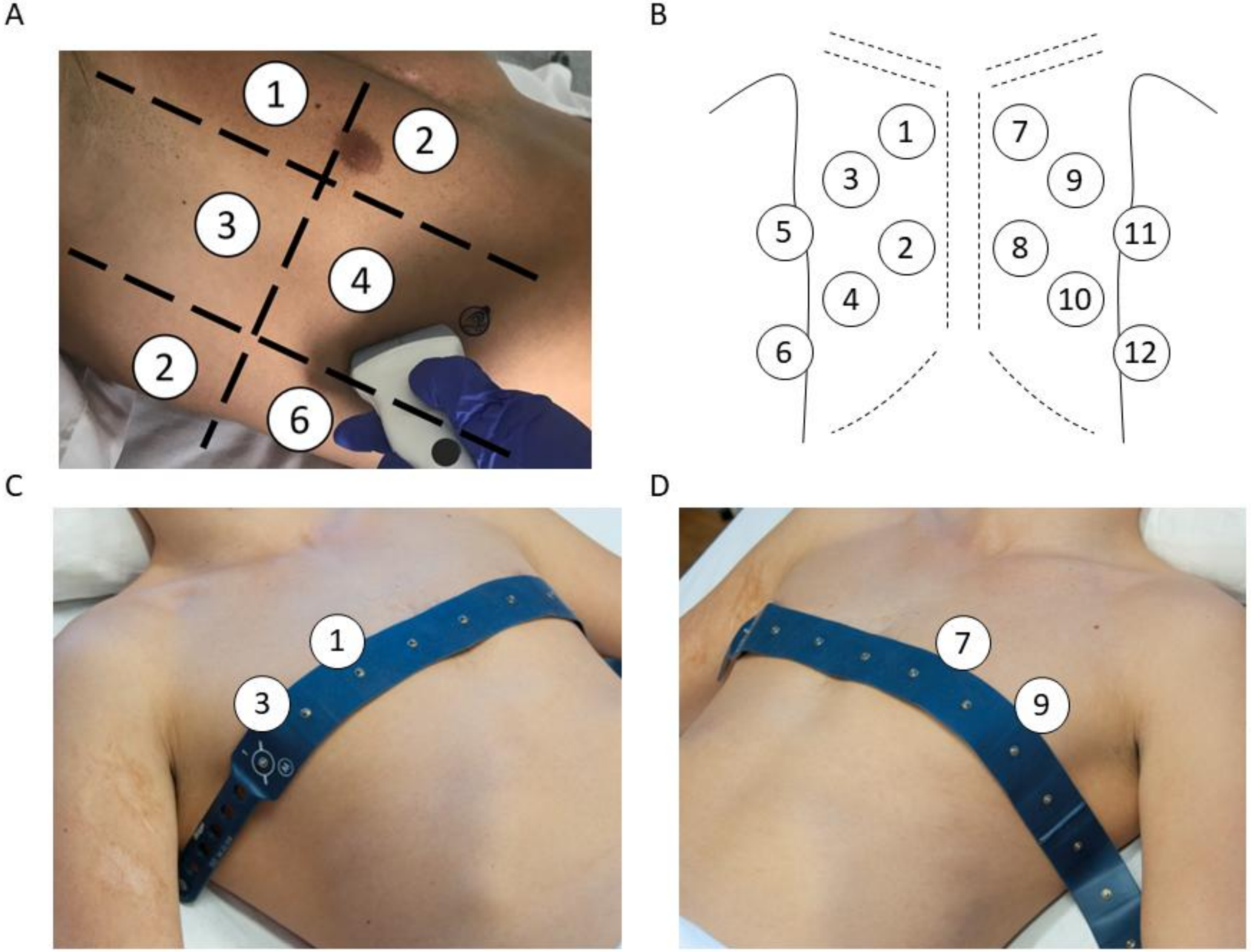

Figure 2S

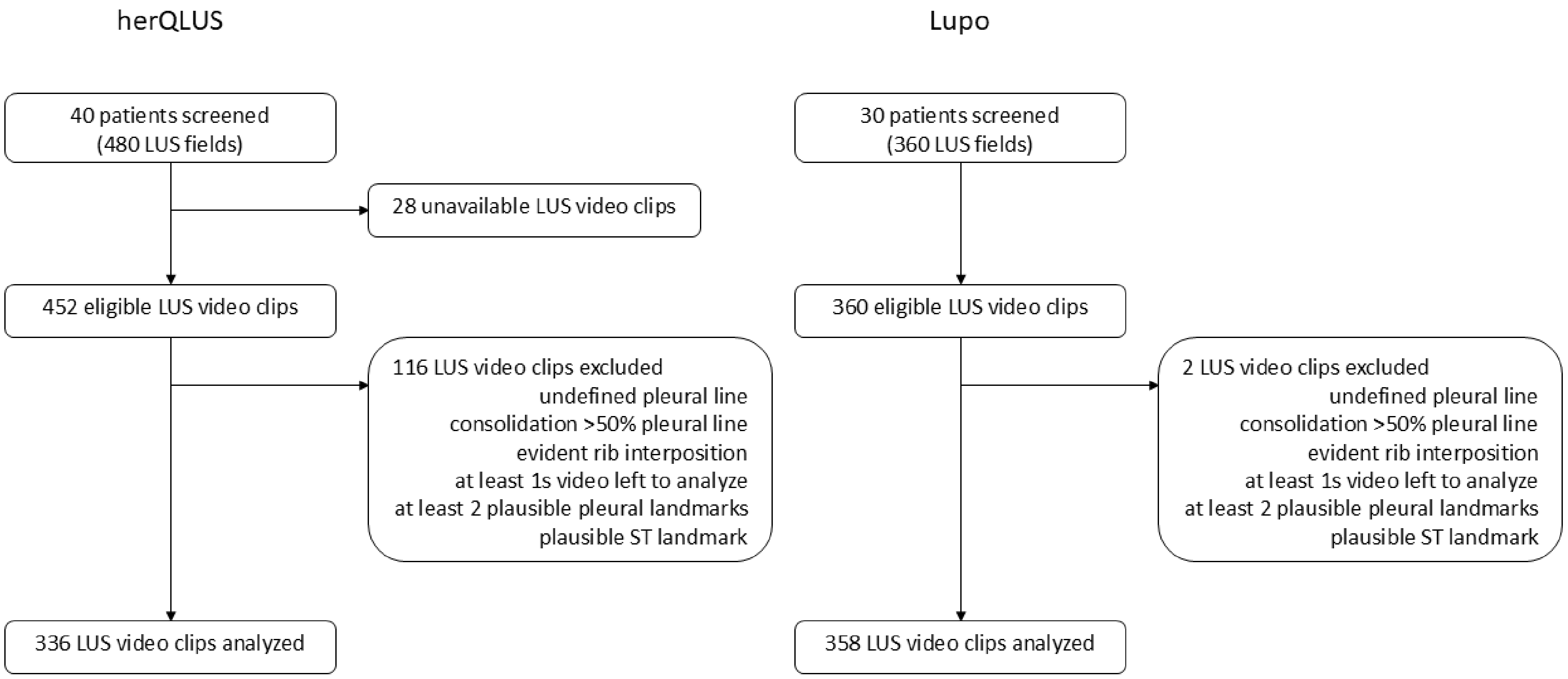
herQLUS
Lupo
40 patients screened
(480 LUS fields)
28 unavailable LUS video clips
452 eligible LUS video clips
116 LUS video clips excluded
undefined pleural line
consolidation >50% pleural line
evident rib interposition
at least 1s video left to analyze
at least 2 plausible pleural landmarks
plausible ST landmark
336 LUS video clips analyzed
30 patients screened
(360 LUS fields)
360 eligible LUS video clips
2 LUS video clips excluded
undefined pleural line
consolidation >50% pleural line
evident rib interposition
at least 1s video left to analyze
at least 2 plausible pleural landmarks
plausible ST landmark
358 LUS video clips analyzed

Figure 3S

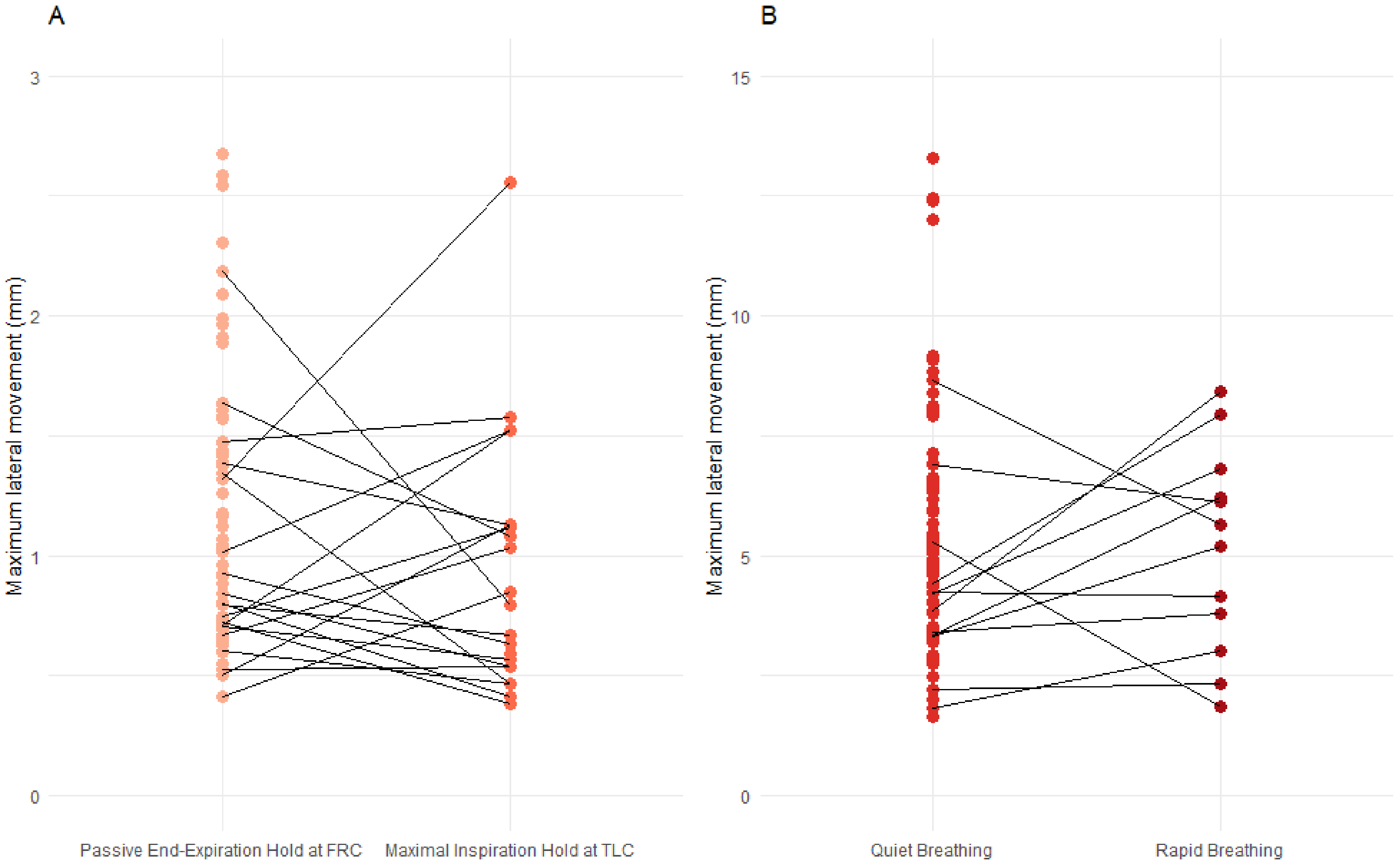
A
B
Maximum lateral movement (mm)
3
2
1
0
Passive End-Expiration Hold at FRC
Maximal Inspiration Hold at TLC
Maximum lateral movement (mm)
15
10
5
0
Quiet Breathing
Rapid Breathing

Figure 4S

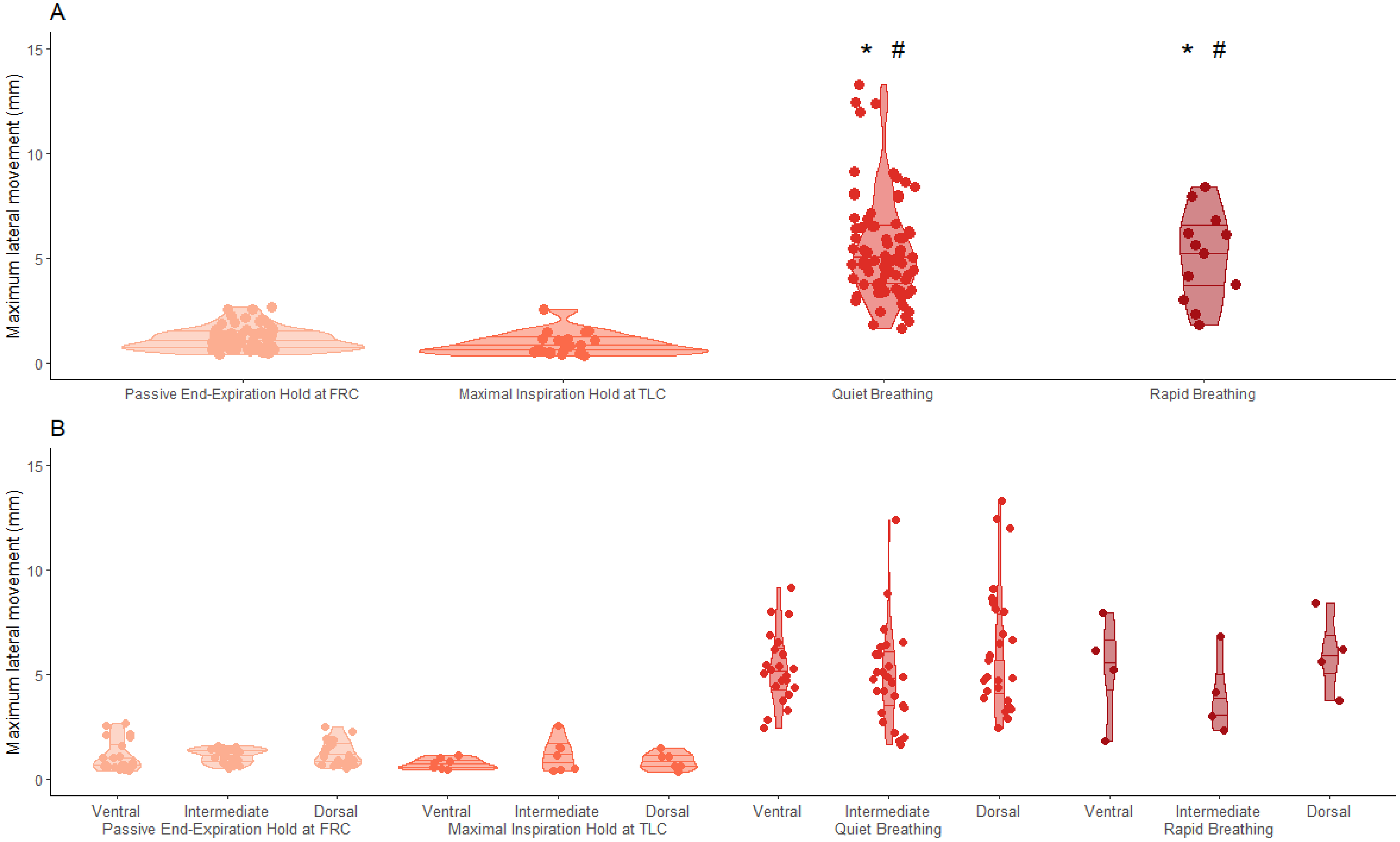

A
Maximum lateral movement (mm)
15
10
5
0
* #
* #
Passive End-Expiration Hold at FRC
Maximal Inspiration Hold at TLC
Quiet Breathing
Rapid Breathing
B
Maximum lateral movement (mm)
15
10
5
0
Ventral
Intermediate
Dorsal
Passive End-Expiration Hold at FRC
Ventral
Intermediate
Dorsal
Maximal Inspiration Hold at TLC
Ventral
Intermediate
Dorsal
Quiet Breathing
Ventral
Intermediate
Dorsal
Rapid Breathing

Figure 5S

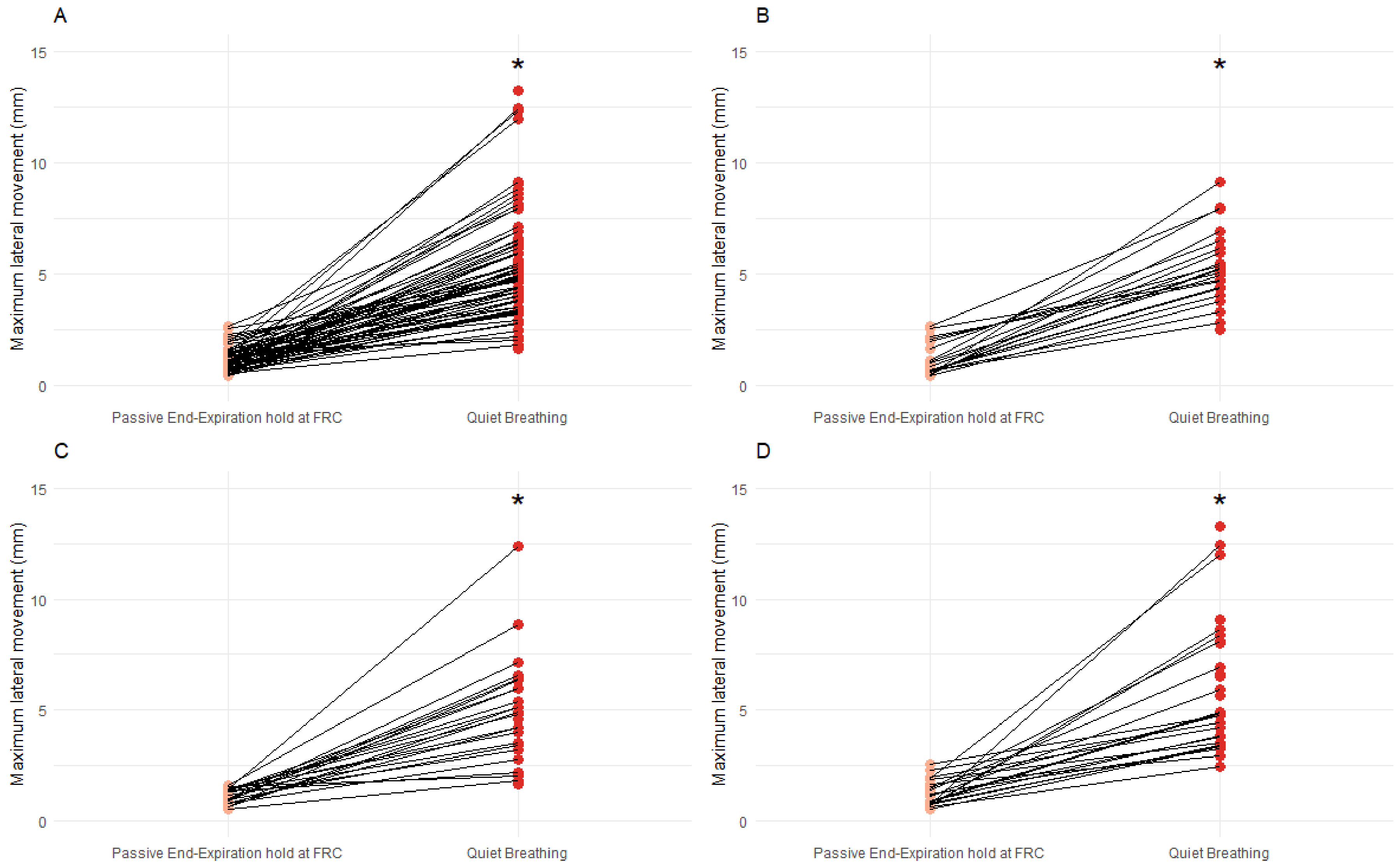
A
B
C
D
Maximum lateral movement (mm)
0
5
10
15
*
Passive End-Expiration hold at FRC
Quiet Breathing

Figure 6S

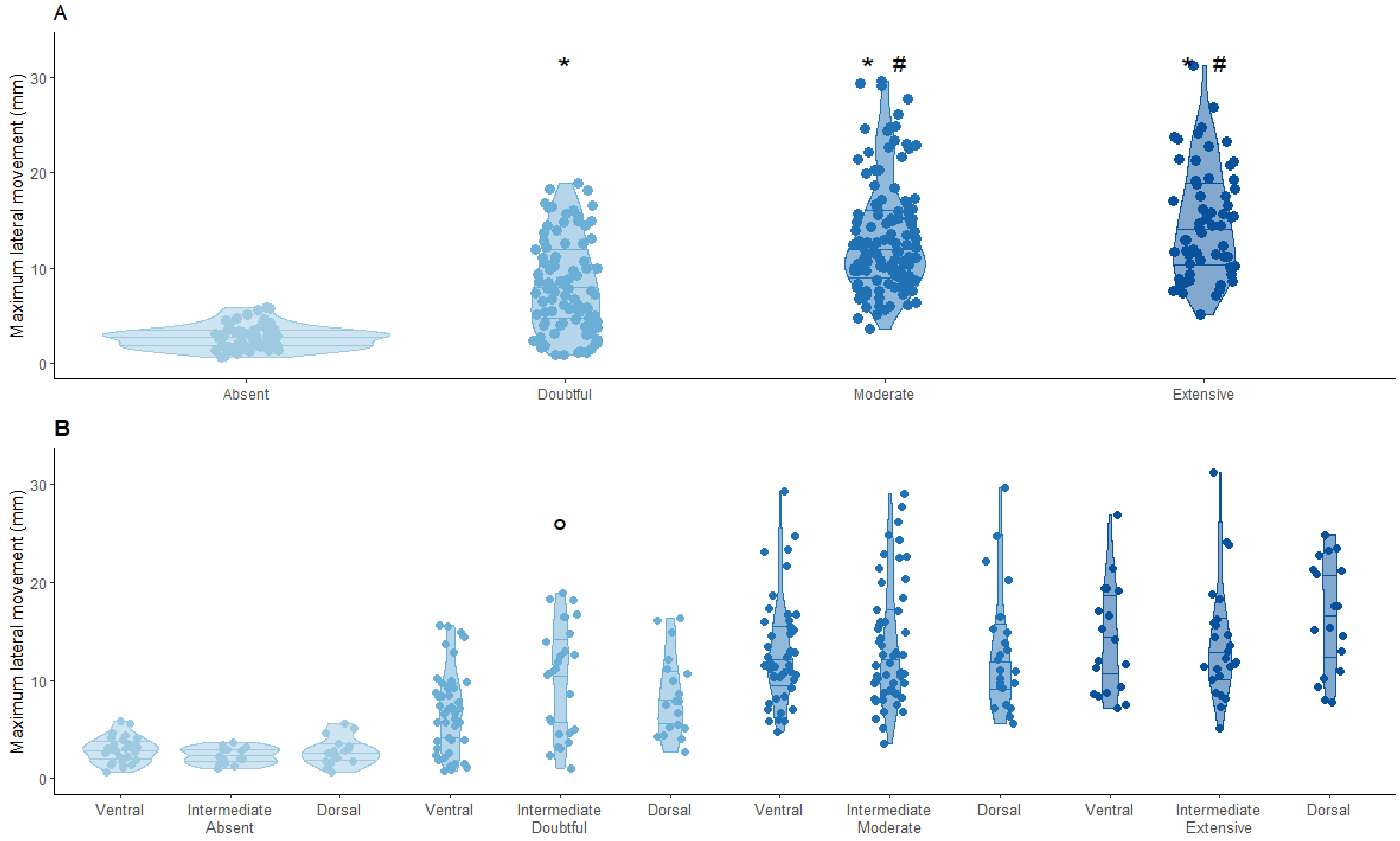
A
Maximum lateral movement (mm)
30
20
10
0
*
* #
* #
Absent
Doubtful
Moderate
Extensive
B
Maximum lateral movement (mm)
30
20
10
0
Ventral
Intermediate
Absent
Dorsal
Ventral
Intermediate
Doubtful
Dorsal
Ventral
Intermediate
Moderate
Dorsal
Ventral
Intermediate
Extensive
Dorsal

Figure 7S

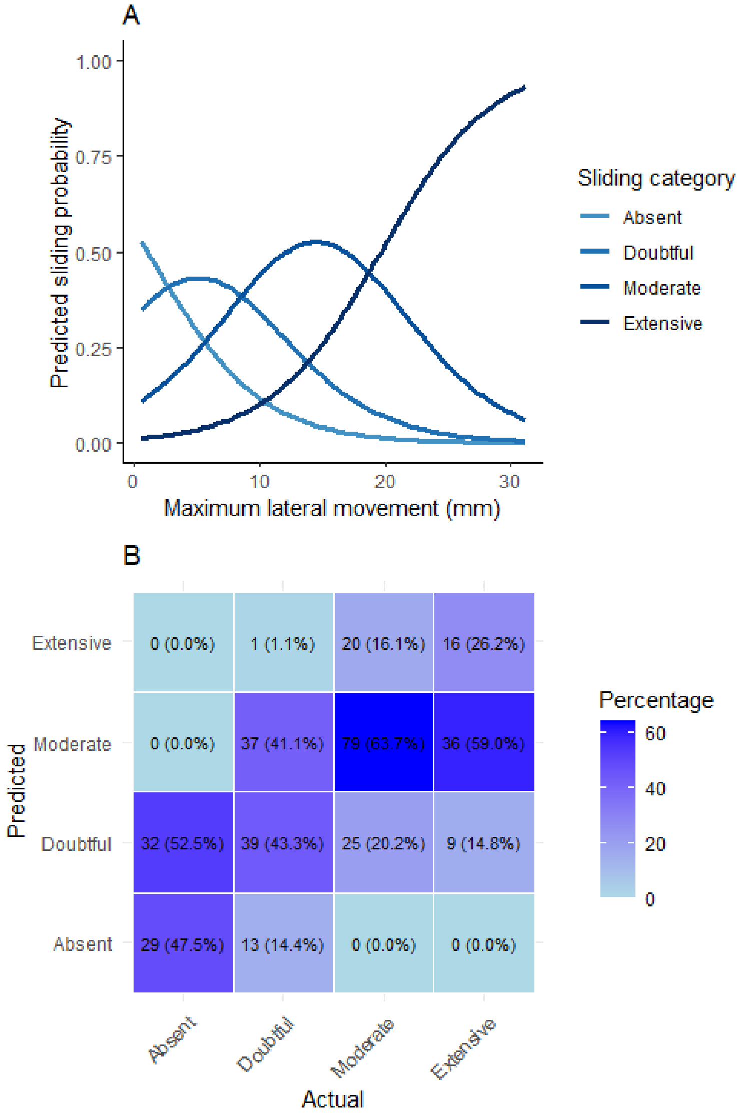
A
Predicted sliding probability
1.00
0.75
0.50
0.25
0.00
0
10
20
30
Maximum lateral movement (mm)
Sliding category
Absent
Doubtful
Moderate
Extensive
B
Predicted
Extensive
Moderate
Doubtful
Absent
0 (0.0%)
1 (1.1%)
20 (16.1%)
16 (26.2%)
0 (0.0%)
37 (41.1%)
79 (63.7%)
36 (59.0%)
32 (52.5%)
39 (43.3%)
25 (20.2%)
9 (14.8%)
29 (47.5%)
13 (14.4%)
0 (0.0%)
0 (0.0%)
Absent
Doubtful
Moderate
Extensive
Actual
Percentage
60
40
20
0

Figure 8S

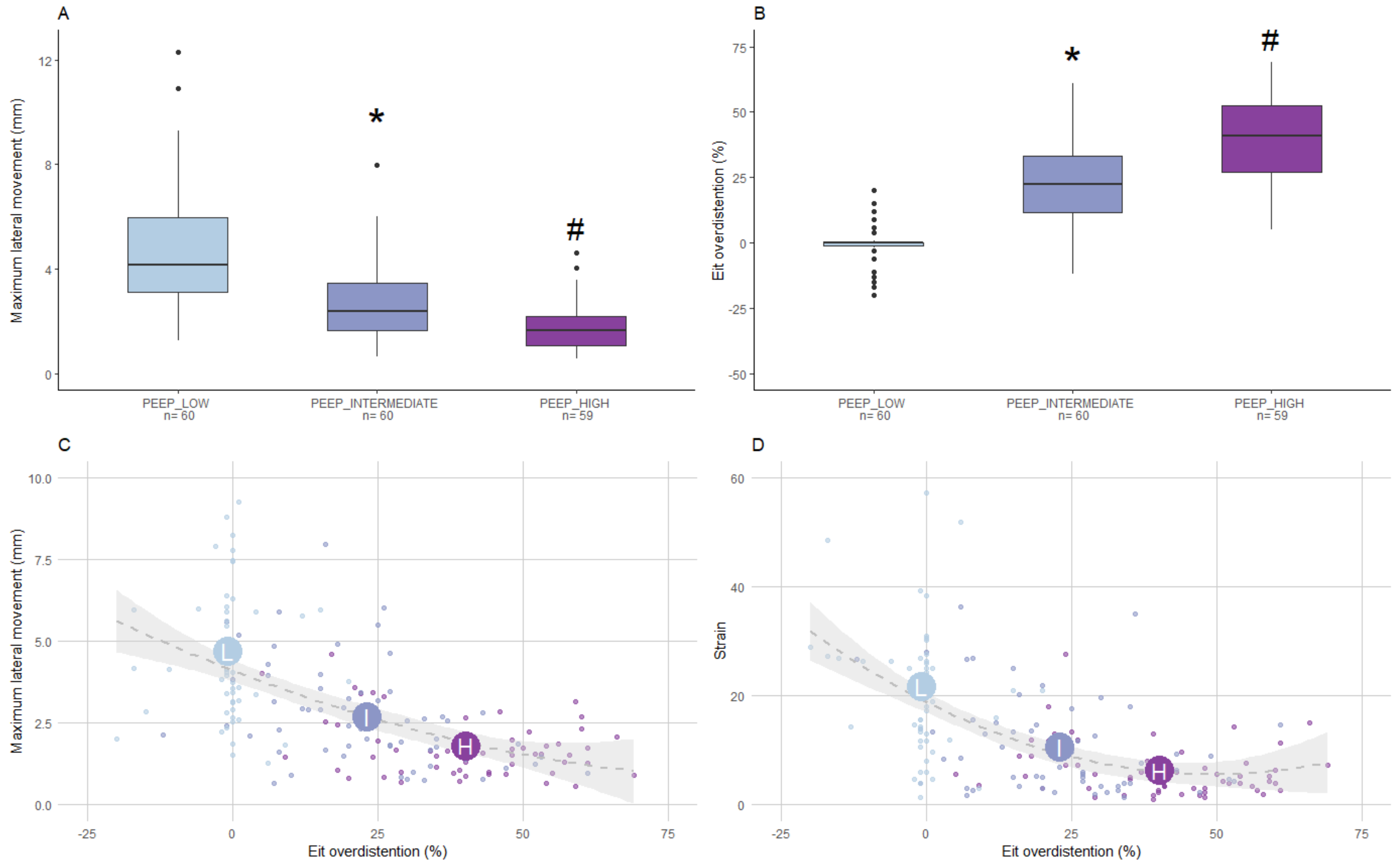

A
Maximum lateral movement (mm)
0
4
8
12
*
#
PEEP_LOW
n= 60
PEEP_INTERMEDIATE
n= 60
PEEP_HIGH
n= 59
B
Eit overdistention (%)
-50
-25
0
25
50
75
*
#
PEEP_LOW
n= 60
PEEP_INTERMEDIATE
n= 60
PEEP_HIGH
n= 59
C
Maximum lateral movement (mm)
0.0
2.5
5.0
7.5
10.0
L
I
H
-25
0
25
50
75
Eit overdistention (%)
D
Strain
0
20
40
60
L
I
H
-25
0
25
50
75
Eit overdistention (%)

Figure 9S

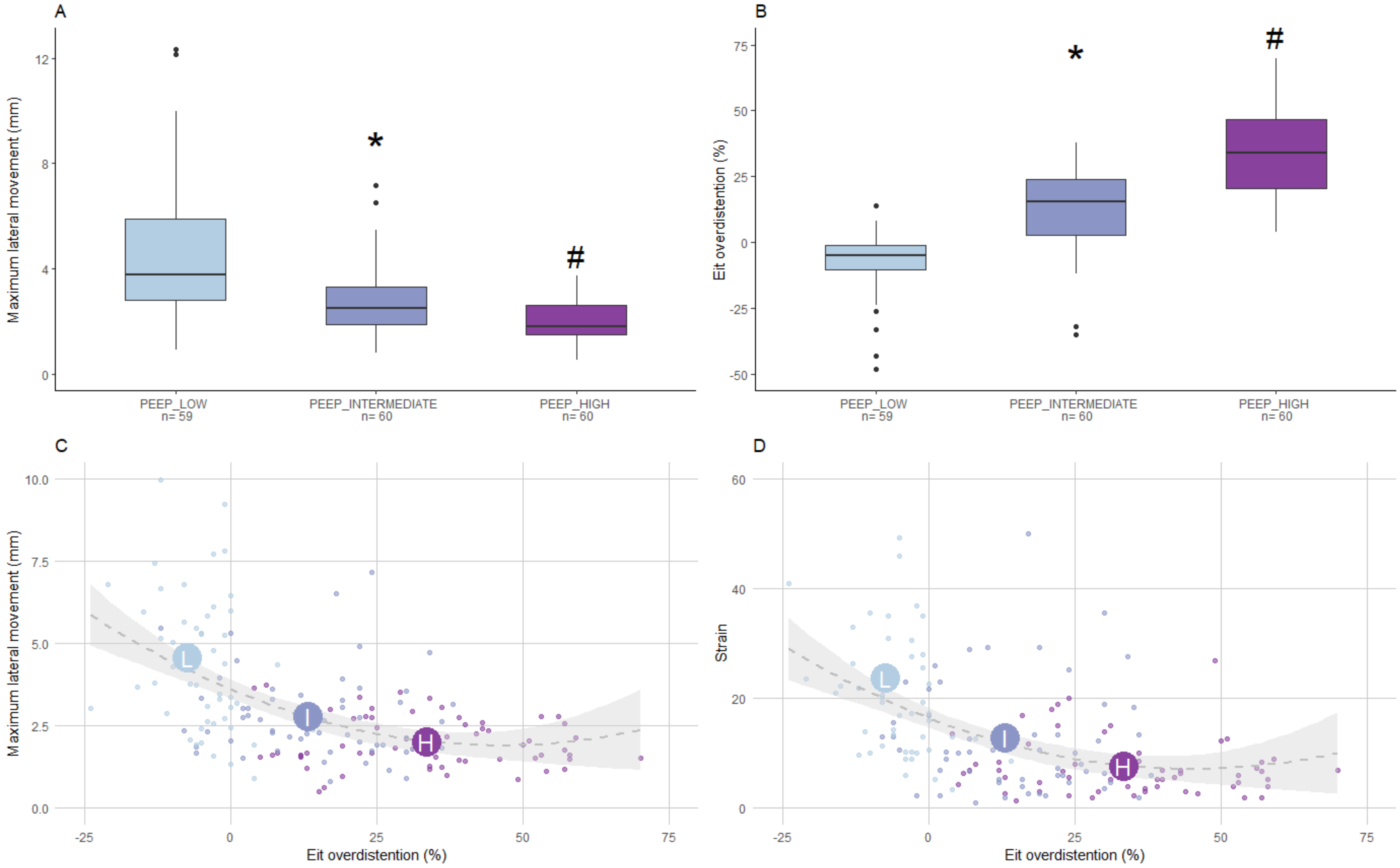

A
Maximum lateral movement (mm)
12
8
4
0
*
#
PEEP_LOW
n= 59
PEEP_INTERMEDIATE
n= 60
PEEP_HIGH
n= 60
B
Eit overdistention (%)
75
50
25
0
-25
-50
*
#
PEEP_LOW
n= 59
PEEP_INTERMEDIATE
n= 60
PEEP_HIGH
n= 60
C
Maximum lateral movement (mm)
10.0
7.5
5.0
2.5
0.0
L
I
H
-25
0
25
50
75
Eit overdistention (%)
D
Strain
60
40
20
0
L
I
H
-25
0
25
50
75
Eit overdistention (%)

Figure 10S

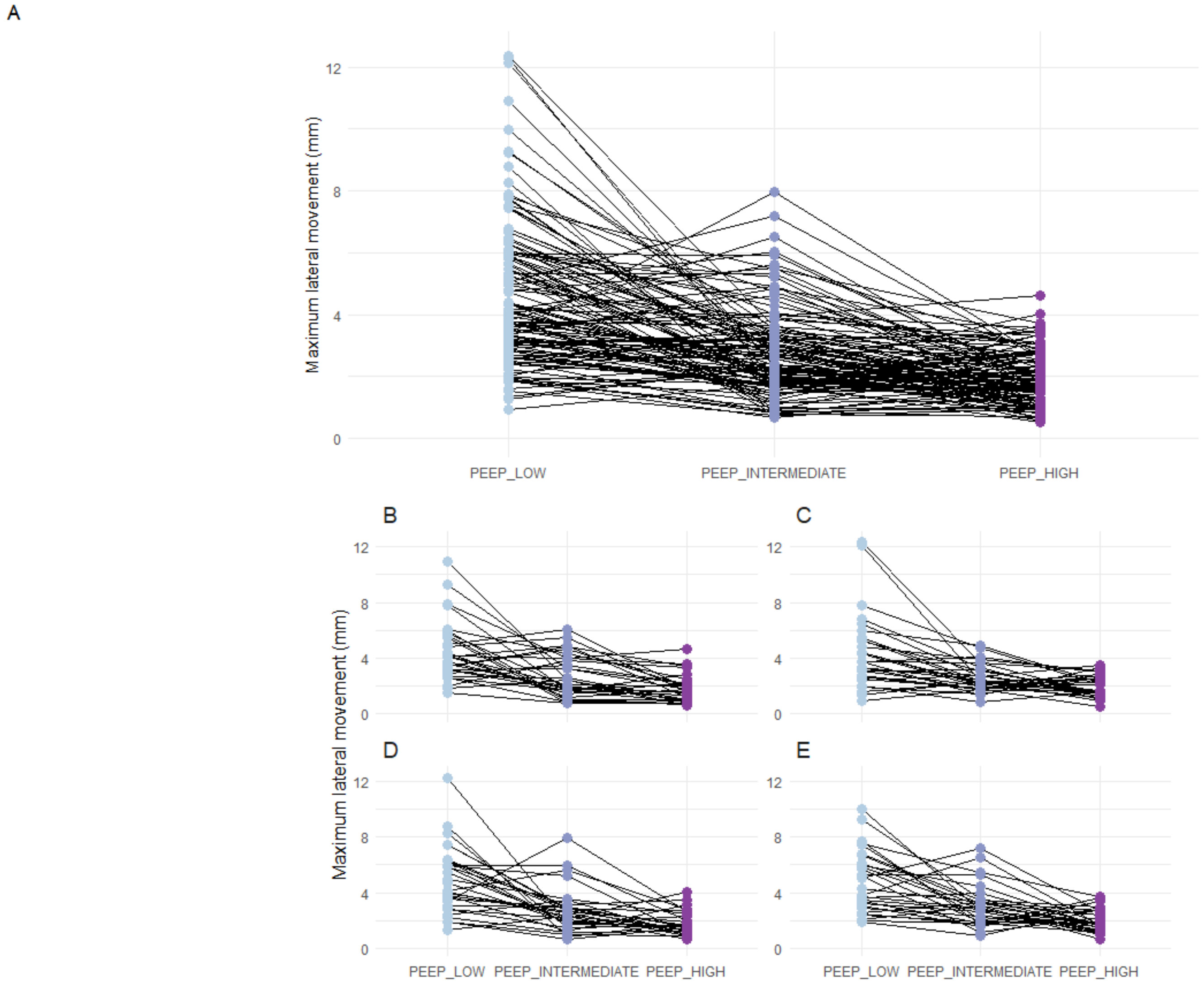
A
B
C
D
E
Maximum lateral movement (mm)
0
4
8
12
PEEP_LOW
PEEP_INTERMEDIATE
PEEP_HIGH
